# Conversion of Gun-Shot Wounds into Incised Wounds as a Means of Speedy Cure

**Published:** 1864-09

**Authors:** J. J. Chisolm

**Affiliations:** P. A. C. S.


ART. X.—*Conversion af Gun-Shot Wounds into Incised Wounds as a means of Speedy Cure.* By Surgeon J. J. CHISOLM, P. A. C. S.

In surgical practice, attention is often called to subcutaneous wounds or injury to the deep structures, accompanied by considerable extravasation of blood, which is a positive sign of crushing of these tissues. In the natural progress of such cases, when the skin has not been much bruised, nature, by her healthy processes, remodels the injured parts, absorbing the effused fluids, and also those portions of tissue caused by the blow and which are no longer filled for use in the economy. Rarely is suppuration induced in such contusions, however extensive they may be, provided the skin remains entire, the natural tendency being to speedy restoration; and very rarely is suppuration, and even sloughing, not induced where the skin is equally crushed with the deeper tissues.

A consideration of these facts, familiar to every practitioner and frequently seen in shell injuries, has suggested to me the propriety of closing the outer orifice of gun-shot wounds, placing them in the condition of subcutaneous injuries, when we should expect such good results as usually follow in the progress of contusions. As soon as possible after the receipt of the injury and before any symptoms of re-action have appeared—having removed all foreign bodies—make two elliptical incisions enclosing the wound, extending through the skin alone; dissect away the lacerated tissues between the elliptical incisions, which will leave two smoothly cut surfaces in lieu of the ragged orifices. These must be brought in perfect opposition by sutures or adhesive straps, and healed by water dressings, so as to ensure union by the first intention. If air can be kept from the track of the ball, although the deep tissues be crushed, experience teaches us that no suppuration will take place; numbers of cases from every battlefield show us that in long circuitous tracks made by balls, when they still remain embedded, with but one orifice permitting the entrance of air, and more especially when the position of the orifice is such that the pressure of a long prominence compresses the track near the outer opening, this orifice rapidly closes and the long sinuous track of crushed tissues gives us further trouble. Simple flesh wounds through a limb, with the free admission of air to the track of the wound, usually requires weeks to effect cicatrization, and sometimes, at the termination of several months, one of the orifices is still discharging, although no foreign body complicates the wound. As long as the wound remains open, it is liable to serious accidents and complications of sloughing, gangrene, hemorrhage, &c., and, when healed, a fibrous, puckered cord represents the track of the ball, tying together the muscles, and intimately connecting the skin with the deeper tissues, so that any movement extended to the cicatrices is painful long after the wound has, to all appearances, healed. Besides, the suppuration induced and the irritation of the muscles perforated, with the diffused effusion of lymph gluing contiguous muscles together, cause contraction and deformity of limbs, which are with difficulty corrected. When the orifices in gun-shot wounds close at an early period, particularly when they heal, as they sometimes do, by a process similar to the first intention, we find no such sequel, and such soldiers very soon return to duty. If the above suggestion could be carried out of converting the contused and lacerated orifices of gun-shot wounds into simple incised wounds, healing readily by the first intention, the mortality after battles would be materially diminished, the hospitals would be rapidly emptied, and the army soon recuperated. If the views suggested as above are correct, and they are supported by numerous instances, there is no necessity of extending the incisions beyond the skin, and as it will be always remembered that the skin immediately around the orifice of entrance sloughs, all this bruised portion must be removed. These simple incisions, extending through the skin alone, can in no case add to the serious character of the wound, nor interfere in any way with the usual progress, should the incision not heal by the first intention. The attention of field surgeons is invited to what an extended experience may prove to be a very simple and satisfactory method of healing rapidly gun-shot wounds.

